# Survival rate of odontogenic descending necrotizing mediastinitis. Our experience in last 5 years

**DOI:** 10.4317/medoral.25585

**Published:** 2022-09-29

**Authors:** Ángela Sada-Urmeneta, Marc Agea-Martínez, Eduardo Monteserín-Martínez, Raúl Antúnez-Conde, Dafne Gascón-Alonso, Gema Arenas-De-Frutos, Carlos Navarro-Cuellar, Ignacio Navarro-Cuellar

**Affiliations:** 1 Oral and Maxillofacial Surgery Department. Hospital General Universitario Gregorio Marañón, Madrid, España

## Abstract

**Background:**

Descending necrotising mediastinitis is one of the most lethal and least frequent forms of mediastinitis. It is a life-threatening infection most frequently originating from an oropharyngeal or odontogenic infection.

**Material and Methods:**

A retrospective study of 6 patients diagnosed and treated for descending necrotising mediastinitis between 2015 and 2020 is reported.

**Results:**

All patients were male, mean age of 34.83 years; 66% were smokers. 83% had an orocervical infection and 34% had initial mediastinal spread. All patients were treated initially with empirical broad-spectrum antibiotics and surgical drainage, with subsequent admission to the Intensive Care Unit; only one of them required tracheostomy. The mean hospital stay was 27.37 days. After a mean follow-up of 6 months, 100% of the cases had a complete recovery.

**Conclusions:**

Early diagnosis and surgical treatment combined with improved life-support treatment in intensive care units and broad-spectrum antibiotic therapy leads to a decrease in associated mortality.

** Key words:**Odontogenic infection, cervical abscess, acute mediastinitis, descending necrotizing mediastinitis, mortality rate.

## Introduction

Mediastinitis is defined as an inflammation or infection in the connective tissue surrounding the mediastinum. One of the most lethal and rarest forms of this clinical entity is descending necrotising mediastinitis (DNM) (1), which originates from an oropharyngeal infection, usually from a tonsillar pillar, or odontogenic infection that spreads through the cervical fascial planes towards the mediastinum.

**Figure 1:**
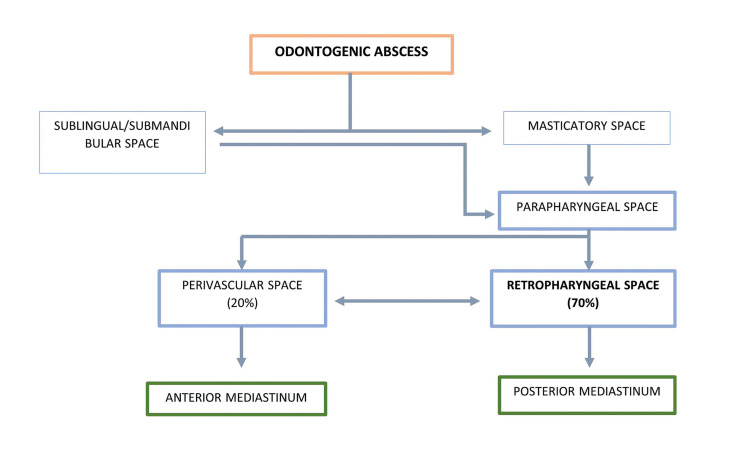
Odontogenic abscess dissemination pathways.

Mediastinal spread from the oro-cervical space occurs through the deep cervical fascia, which has 3 lamellae (pretracheal, visceral and prevertebral) delimiting the cervical spaces (pretracheal, perivascular and prevertebral or retropharyngeal) through which the infection advances (2-6) (Fig. 1).

DNM was first reported by Pearse in 1938 (2). However, the current concept of mediastinitis was not est<ablished until 1983, when Estrera *et al*. defined the diagnostic criteria for this disease (7) (Table 1).

DNM is a polymicrobial infection, in which both aerobic and anaerobic germs are isolated. The most frequent cause is an odontogenic focus, with the second or third mandibular molar as the most common origin. It is most seen in middle-aged men, with increased risk in cases of impaired immunity, nutritional deficiencies, patients with poor oral hygiene and smokers.

This clinical entity usually begins as an odontogenic infection with local pain and swelling; and fever above 38°C is part of the initial symptoms in about half of patients. Other complicating symptoms such as odynodysphagia, respiratory distress and chest pain can progressively appear.

Early clinical diagnosis is crucial in the treatment of this disease and must be supported by blood and imaging tests. Cervical and thoracic CT scan is the gold standard radiological test for the diagnosis of this condition. It is important to consider the anatomical classification proposed by Endo *et al* (8,9), as it could mark the access route for surgical drainage and the prognosis of this entity. Type I or focal is that which is confined to the upper mediastinum, above the carina. Type II, or diffuse, can be subdivided into IIa (anterior mediastinum) or IIb (posterior mediastinum).

The clinical progression of this infection is both fast and aggressive, and the mortality rates reported are high, ranging from 11% to 40% (3,10). Therefore, early treatment is necessary, with patient stabilization, empirical broad-spectrum antibiotic therapy, surgical drainage and lifesupport in intensive care units (ICU).

## Material and Methods

A retrospective descriptive study of a series of cases, from 1 January 2015 to 31 December 2020, by the Oral and Maxillofacial Surgery Department of the Hospital General Universitario Gregorio Marañon was carried out.

The aim of this study is to describe our experience in DNM of odontogenic cause in the last 5 years at our center, and to assess whether there is a change in the trend of associated mortality regarding to the those reported in the literature.

**Table 1:**
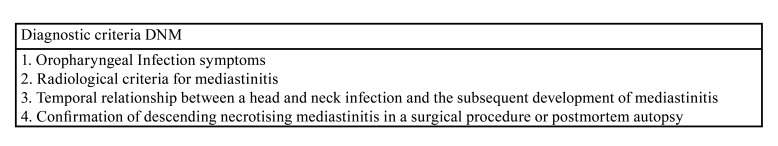
Odontogenic abscess dissemination pathways.

The variables studied were age, gender, risk factors (smoking, immune disorders), clinical debut, odontogenic source, empirical and specific antibiotic treatment, surgical interventions, need of surgical tracheostomy, ICU hospitalization, days of hospital admission, follow-up, and mortality.

The following inclusion criteria were established:

1) Oro-cervical infection of odontogenic origin that subsequently developed mediastinal infection, with space-time relationship between both entities.

2) Patients over 18 years of age.

3) Cervico-thoracic CT scan as diagnostic imaging test.

4) Need for surgical procedure for drainage of collections.

5) Sample collection. Culture and antibiogram, following the measures established by the Microbiology Department of our center.

Exclusion criteria were:

1) Other descending necrotizing mediastinitis different from odontogenic cause 

2) Failure to find information on some of the variables studied.

A total of 132 patients with a diagnosis of odontogenic abscess requiring hospital admission were selected, of which 6 were complicated by subsequent mediastinitis and met the inclusion criteria.

## Results

All patients were male, ranged in age from 22 to 48, with a mean of 34.83 years old. None of the patients had drug allergies or impaired immunity. 66% of the sample were active smokers (Table 2).

In all patients the cause of the odontogenic foci was the 2nd or 3rd lower molar. 2 of the cases had undergone a mandibular molar extraction in the previous days, without subsequent antibiotic therapy.

The most frequent initial symptom in our series was odontalgia of several days of evolution, with progressive swelling at the para- and/or submandibular region. Other less common associated symptoms and signs were trismus, odynophagia with or without dysphagia and floor of mouth swelling.

All patients underwent a blood test and cervical CT scan with intravenous contrast as initial diagnostic studies. All blood tests showed inflammatory parameters such as leukocytosis with neutrophilia and elevated C-reactive protein (C-reactive protein).

In 83% of the patients (5 cases), orocervical infection with organized collection was present. In the other patient, an orocervical cellulitis without obvious collection was diagnosed. In 33% (2 patients) of the patients, initial mediastinal extension was also noted, and a thoracic CT scan was requested. (Table 3).

**Table 2:**
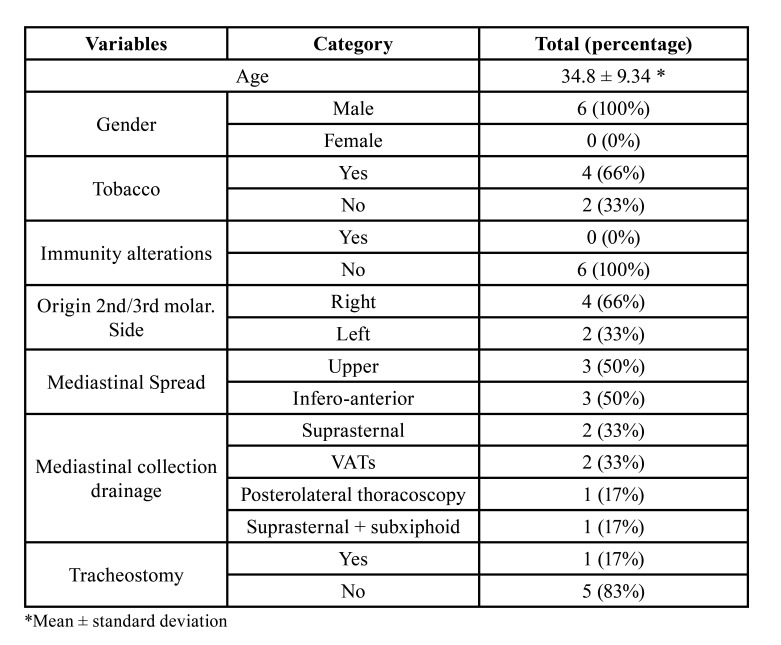
Summary of cases.

**Table 3:**
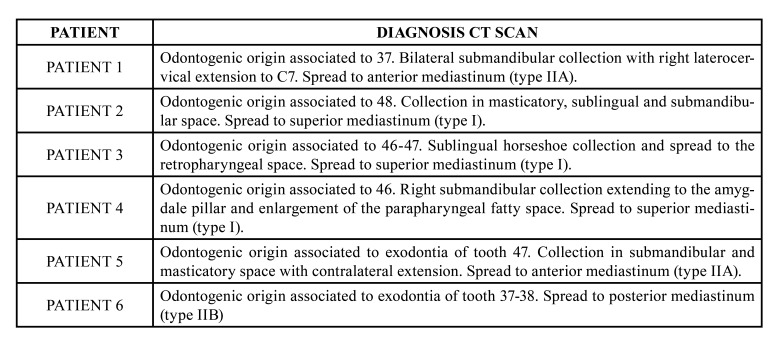
CT localisation of early orocervicomediastinal infections.

All patients were hospitalized, empirical broad-spectrum intravenous antibiotics were prescribed, and surgery was considered. 83% were firstly treated with amoxicillin-clavulanic Acid 2g/200mg 8-hourly, and 17% with meropenem 1g 12-hourly and clindamycin 600mg 8-hourly, as the last patient had taken a cycle of antibiotics with amoxicillin-clavulanic acid 875/125mg 8-hourly.

In 66% of the patients (4 patients), drainage of the orocervical collection and exodontia of the causal teeth was performed under general anesthesia. 34% (2 patients) of the cases were also initially treated by the Hospital's Thoracic Surgery Department (combined with cervical drainage and treatment of the odontogenic cause) due to the mediastinal extension of their infection. The other mediastinal drainage procedures (66%) were performed on a second surgical time due to the infection's progression.

1 patient (17%) underwent tooth extraction under local anesthesia and the other one (17%) was closely monitored, as 2 molars had been previously extracted and no collection was visible in the diagnostic CT scan.

Reintervention was required in 83% of patients due to disease progression, with both clinical and radiological worsening (Table 4).

In all patients, microbiological cultures were obtained and subsequently analyzed by the Hospital's Microbiology department. Aerobic and anaerobic microorganisms were isolated; the most frequently isolated germ was *Streptococcus* viridans, present in 100% of the samples. The second most common microorganism was *Candida albicans* (34%); other microorganisms found in the samples were Actynomices odontolyticus, Finegoldia magna, Eubacterium lentum, *Prevotella* denticola, *Prevotella* bucae. Antibiogram was performed on all samples and treatment was adjusted according to antibiotic sensitivity. The next antibiotic combinations were used: meropenem 1g 8-hourly plus linezolid 600mg 12-hourly, meropenem 1h 8-hourly plus clindamycin 600mg 8-hourly, meropenem 1g 8-hourly plus vancomycin 1g 8-hourly, fluconazole 400mg 24-hourly plus erythromycin 150mg 6-hourly, piperaziline-tazobactam 4g 8-hourly, meropenem 1g 8-hourly plus fluconazole 400mg 24-hourly.

**Figure 2:**
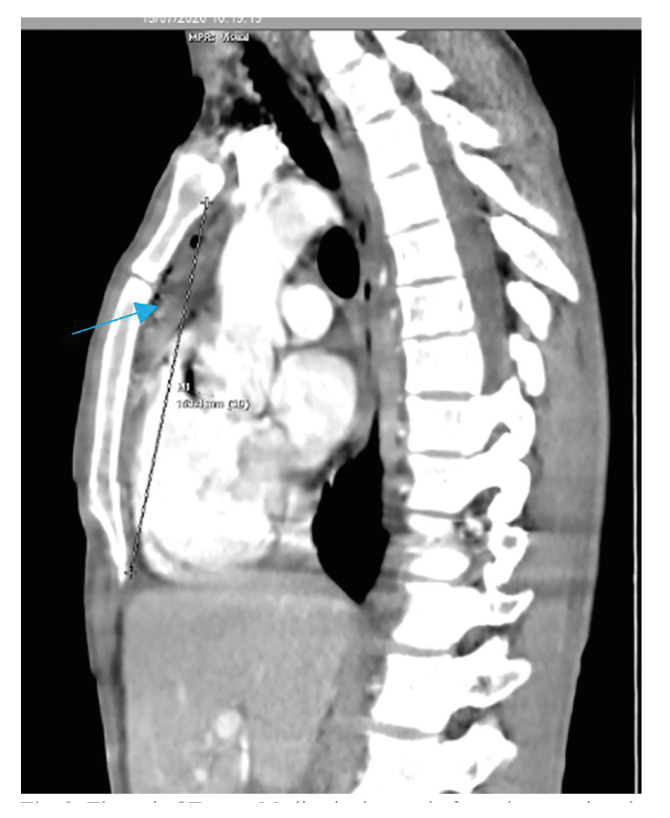
Thoracic CT scan. Mediastinal spread of an odontogenic collection (Blue Arrow).

**Figure 3:**
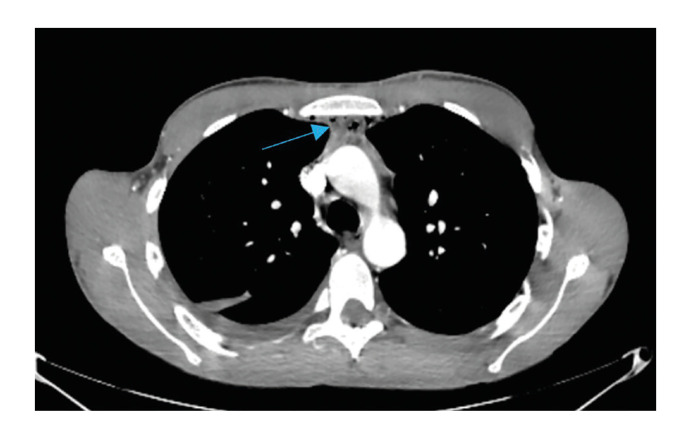
Thoracic CT scan. Mediastinal spread of an odontogenic collection (Blue Arrow).

In the first postoperative days, 34% (n=2) of patients presented with chest pain associated with dyspnea and desaturation. An additional 34% (n=2) of patients developed pain and local deterioration. In all of them, progression of the infection towards the mediastinum was suspected by mediastinal widening in simple thoracic x-ray, and cervico-thoracic CT with intravenous contrast was done to verify the clinical suspicion. (Fig. 2, Fig. 3).

100% of patients required admission to the Intensive Care Unit, requiring invasive mechanical ventilation, with a mean stay of 12 days. Tracheostomy was necessary in 17% (n=1) of them.

During admission, 66% (n=4) of patients were diagnosed with one of the following complications: bacteremia, oral candidiasis, acute hepatitis, atelectasis, and repeated syncope of non-cardiogenic cause.

The mean length of hospital admission was 27.17 days, ranging from 17 to 42 days.

All patients were discharged from hospital and were followed up for a mean of 6 months (1-11 months) by the Thoracic and Maxillofacial Surgery Departments, with no deaths at the end of the study.

**Table 4:**
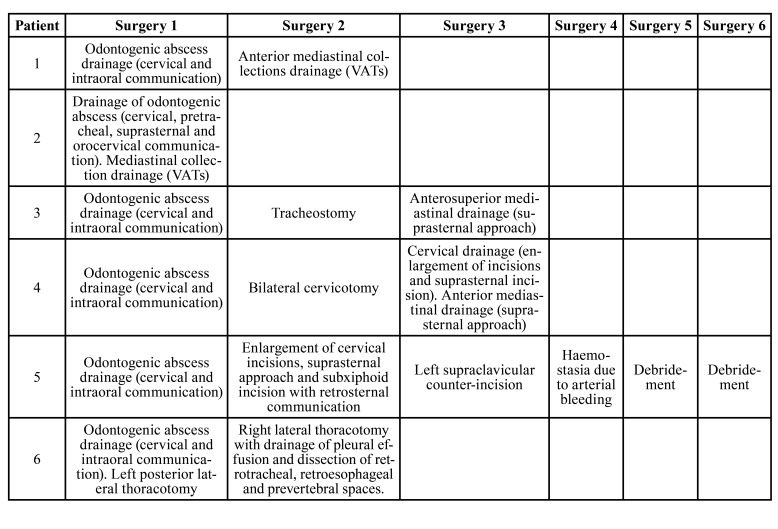
Surgical procedures.

## Discussion

DNM is a rare polymicrobial infection originated by aerobic and anaerobic microorganisms, which can cause tissue necrosis, perpetuate a suiTable environment for anaerobic microorganisms and lead to cervical fascial plane dissection with subsequent dissemination towards the mediastinum (6,11). The origin of this condition is usually related to infectious diseases related to the second and third mandibular molars (11,12). In all patients of this research, the initial etiology of DNM was a pathology associated to the second or third inferior molars.

The most frequent route way of dissemination of DNM is through the retropharyngeal space (70%), which communicates with the posterior mediastinum, followed by the perivascular space (20%, concerning the anterior or middle mediastinum). The remaining 10% of infectious processes progress through the pretracheal space, also causing involvement of the anterior or middle mediastinum (5). The mediastinal space affected may therefore be determined by the location of the initial infection. In Table 2 we can see the initial local infection and the mediastinal spread.

The diagnosis of this clinical entity is based on laboratory tests (notably leukocytosis with neutrophilia), elevated acute phase reactants such as CRP and fibrinogen, and radiological imaging test, with cervico-thoracic CT with intravenous contrast being the test of choice. All our patients underwent an initial blood test, in which leukocytosis with neutrophilia and elevated other inflammatory parameters were observed, as well as a cervical CT scan on admission to our center. Thoracic CT scan should be performed initially if mediastinal spread is suspected, for example, in case of dyspnea, chest pain, pathological cardiopulmonary auscultation, ... Only one patient required a thoracic CT scan on admission to the emergency department. In the remaining patients a chest scan was performed during admission due to clinical worsening.

Treatment should be early and aggressive, based on tooth extraction (if it has not been already done), surgical drainage, exhaustive irrigation of oro-cervical and mediastinal collections (11) and broad-spectrum intravenous antimicrobial treatment, which can later be adjusted according to the antibiogram results. Patient must be admitted to ICU for further life-sustaining treatment. In our sample, all patients underwent surgical tooth extraction if necessary and surgical drainage and debridement (cervical and thoracic, in one or multiple procedures depending on patients' evolution.). 100% of them were admitted to ICU and started on empirical broad-spectrum antibiotic therapy, which was subsequently adjusted according to the antibiogram results.

When deciding the mediastinal drainage surgical approach, it is important to consider the anatomical classification proposed by Endo *et al* (8,9). Thus, in type I, a cervical/suprasternal approach may be adequate. In type II, however, a posterolateral thoracotomy might be necessary (5,13).

In recent studies, videothoracoscopic drainage (VATS) has gained importance (3,8,14), which allows faster patient recovery, as it is a less aggressive technique. All patients in our series required drainage of cervical and mediastinal collections (33% suprasternal approach, 33% by VATS, 17% right posterolateral thoracotomy and the other 17% suprasternal and subxiphoid approach).

Some authors consider that prophylactic tracheostomy is a good option in the management of the patient's airway (13,15,16). However, others argue that it could contribute to the persistence of cervical and mediastinal infection by allowing aspiration of purulent material (5,17). Only 17% of our patients required surgical tracheostomy during their stay in the Intensive Care Unit for better airway management, as the patient may need a prolonged intubation

Mortality in DNM is high, ranging from 11% to 40%, despite early treatment. Pearse described a 35% mortality in patients who had undergone surgical treatment, compared to 85% mortality in patients in whom drainage of collections was not performed (2). Other authors describe lower but high mortality rates in their respective series. Mistosh *et al*. reported an overall mortality in their series of 33,3% (18) and Ridder *et al*. reported a 11,1% of mortality rate (10). The high mortality of this entity may be explained by a delay in diagnosis and treatment, which would lead to dissemination of the infection with possible multi-organ failure. In this descriptive study, there were no deaths, which could be associated to an early diagnosis; anatomical localization, as in our series the most frequently observed type of mediastinitis was type I (upper mediastinum); an aggressive treatment, and a proper patient management in ICU.

## Conclusions

DNM is a rare clinical entity with a potentially high mortality rate that requires early diagnosis and treatment.

 Diagnosis should be based on analytical and imaging studies, with contrast-enhanced cervicothoracic CT being the CT scan of choice.

Aggressive treatment based on broad-spectrum antibiotic therapy, surgical drainage, and good management of life support measures in intensive care units may lead to a decrease in mortality of this disease.
